# Cellular heterogeneity of circulating CD4^+^CD8^+^ double-positive T cells characterized by single-cell RNA sequencing

**DOI:** 10.1038/s41598-021-03013-4

**Published:** 2021-12-08

**Authors:** Sung Min Choi, Hi Jung Park, Eun A. Choi, Kyeong Cheon Jung, Jae Il Lee

**Affiliations:** 1 Graduate Course of Translational Medicine, Seoul National University College of Medicine, Seoul, 03080 Republic of Korea; 2 Transplantation Research Institute, Seoul National University College of Medicine, Seoul, 03080 Republic of Korea; 3 Department of Pathology, Seoul National University College of Medicine, Seoul, 03080 Republic of Korea; 4 Integrated Major in Innovative Medical Science, Seoul National University Graduate School, Seoul, 03080 Republic of Korea; 5 Department of Medicine, Seoul National University College of Medicine, Seoul, 03080 Republic of Korea

**Keywords:** Lymphocytes, Translational immunology, High-throughput screening

## Abstract

Circulating CD4^+^CD8^+^ double-positive (DP) T cells are associated with a variety of disease states. However, unlike conventional T cells, the composition of this population is poorly understood. Here, we used single-cell RNA sequencing (scRNA-seq) to analyze the composition and characteristics of the DP T cell population circulating in the peripheral blood of cynomolgus monkeys. We found that circulating DP T cells not only contain a large number of naïve cells, but also comprise a heterogeneous population (CD4 CTL-, Eomes^+^ Tr1-, Th2-, Th17-, Tfh-, Treg-, CD8 CTL-, and innate-like cells) with multiple potential functions. Flow cytometry analysis revealed that a substantial number of the naïve DP T cells expressed CD8αβ, as well as CD8αα, along with high expression of CD31. Moreover, the CD4^hi^CD8^lo^ and CD4^hi^CD8^hi^ populations, which express high levels of the CD4 coreceptor, comprised subsets characterized by helper and regulatory functions, some of which also exhibited cytotoxic functions. By contrast, the CD4^lo^CD8^hi^ population with high CD8 coreceptor expression comprised a subset characterized by CD8 CTL- and innate-like properties. Taken together, the data show that scRNA-seq analysis identified a more diverse subset of the circulating DP cells than is currently known, despite this population being very small.

## Introduction

The unbiased and high-throughput nature of modern single-cell RNA sequencing (scRNA-seq) approaches has proven invaluable for describing heterogeneous cell populations^1,2^. Furthermore, cellular indexing of transcriptomes and epitopes by sequencing, a method in which oligonucleotide-labeled antibodies are used to integrate cellular protein and transcriptome measurements into an efficient single-cell readout, was described recently^3^. This process provides a detailed understanding of limited T cell frequencies at the single-cell level, while also being able to reveal the broad heterogeneity of a specific T cell subset.

In addition to CD4 or CD8 single-positive (SP) T cells, which originate in the thymus, where they also commit to a certain lineage and undergo positive or negative selection, CD4^+^CD8^+^ double-positive (DP) T cells that express both CD4 and CD8 co-receptors simultaneously are present in peripheral blood and tissues (e.g., lymph nodes, liver, and spleen)^4,5^. Many reports reveal that this population is associated with several pathological conditions, including cancer^6,7^, autoimmune disorders^8^, graft rejection^4^, and viral infections^9,10^. In most cases, these DP T cells are found in abundance in the blood and/or target organs, suggesting that they are involved in the pathological process. Therefore, peripheral DP T cells may be either differentiated effector T cells or resting memory T cells derived from CD4^+^ or CD8^+^ T cells in response to chronic inflammatory conditions^10,11^. Alternatively, it may be that this population has regulatory properties^12^, or follicular helper^13^ or innate and adaptive functions^14^.

The various properties and functions of conventional T cells are defined according to expression of various cell surface markers and receptors following exposure to antigens or pathogens; alternatively, they are classified generally according to whether they secrete specific cytokines or effector molecules. Nevertheless, the phenotypic properties and functional roles of circulating DP T lymphocytes have not been characterized completely. For instance, in a previous study^4^, we found that some DP T cells express significant levels of promyelocytic leukemia zinc finger (PLZF)^15^ and eomesodermin (Eomes)^16^, both markers of innate lymphocytes, when compared with CD4 SP and CD8 SP T cells. In this respect, DP T cells may be another type of innate-like T cell and so may be considered to be cells with similar characteristics.

To date, most detailed studies of conventional CD4^+^ or CD8^+^ T cells have focused on their immunological functions, or have been conducted in the context of small populations such as NKT, MAIT, and γδT cells. Although CD4^+^CD8^+^ DP T cells can be detected easily in peripheral blood, their role, function, and biological significance are poorly understood. Here, we used the scRNA-seq platform to analyze the characteristics of circulating DP T cells and to compare their transcriptomic profiles with their cellular phenotypes and cytokine secretion patterns.

## Results

### Heterogeneity within the CD4^+^CD8^+^ DP T cell population

**Figure 1:**
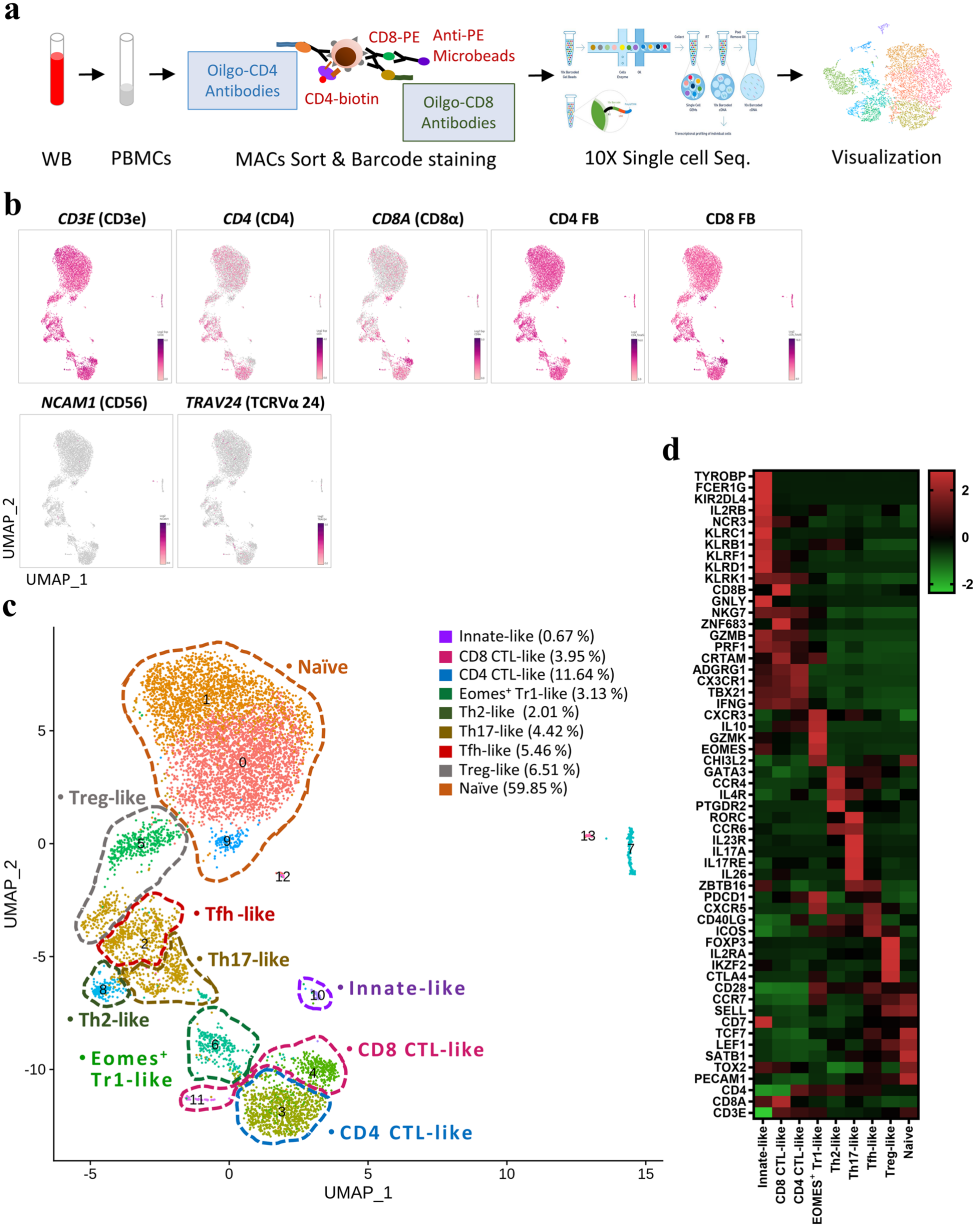
Single-cell RNA-seq analysis of CD4^+^CD8^+^ DP T cells. (**a**) Schematic showing the procedures used for cell isolation, CD4 and CD8α antibody-derived tag labeling, and single-cell RNA analysis of CD4^+^CD8^+^ DP T cells from cynomolgus monkeys. (**b**) UMAP representing *CD3E*, *CD4*, *CD8A*, CD4 FB, CD8 FB, *NCAM1* (CD56), and *TRAV24* (TCRVα24) expression by CD4^+^CD8^+^ DP T cells. (**c**) Transcriptome-based clustering of the single-cell expression profiles of DP T cells revealed 14 distinct clusters (numbered). After filtering, nine cell types (dotted lines) were defined according to expression of marker genes (cell composition). (**d**) Heatmap showing relative expression of marker genes representing the nine DP subpopulations; shown are the Z-scores obtained by differentially expressed gene (DEG) analysis of all defined subpopulations. The heatmap was generated using GraphPad Prism 8.0.2. The number in UMAP indicates the cluster number. *FB* feature barcode.

To characterize circulating DP T cells, we first isolated CD4^+^CD8^+^ DP T cells from the peripheral blood of healthy adult monkeys using MACS microbeads. Sorted DP T cells were then incubated with CD4 and CD8α antibody-derived tags (ADTs), a feature barcode. We then ran the 10× Genomics single-cell workflow (Fig. 1a). After filtering, we analyzed 8338 cells, detecting a total of 13,738 genes (Supplementary Fig. S1). A uniform manifold approximation and projection (UMAP) of sorted DP T cells showed that *CD3E*, CD4 FB, and CD8 FB were highly expressed by all clusters, whereas *NCAM1* (CD56) and *TRAV24* (TCRVα24) were expressed at either very low levels or were absent (Fig. 1b). In other words, scRNA-seq analyzed CD4^+^CD8^+^ DP T cells, not NK or NKT cells. The 14 clusters classified using the nearest neighbor algorithm of Seurat analysis are defined as nine cell types by differential expression and UMAP coordinates for specific marker genes (Fig. 1c,d): a CD4 CTL-like cluster expressing *CD4*, *KLRK1*, *PRF1*, *GZMB*, *CRTAM*, and *TBX21*; an Eomes^+^ type 1 regulatory T (Tr1)-like cluster expressing *EOMES*, *GZMK*, and *CHI3L2* but lacking the canonical Treg markers *IL2R* and *FOXP3*^17^; a Th2-like cluster expressing *GATA3*, *CCR4*, *PTGDR2*, and *IL4R*; a Th17-like cluster expressing *RORC*, *CCR6*, *IL17A*, *IL23R*, *KLRB1*, and *ZBTB16*; a circulating Tfh-like cluster expressing *CXCR5*, *PDCD1*, *ICOS*, *CD40LG, CCR7*, and *SELL*; a distinct regulatory T cell (Treg)-like cluster expressing Treg-defining genes *FOXP3*, *IL2RA*, *IKZF2*, and *CTLA4*; a CD8 CTL-like cluster expressing *CD8A*, *KLRD1*, *KLRK1*, *CRTAM*, *ZNF683*, *PRF1*, and *GZMB*; an innate-like cluster expressing *KLRC1*, *KLRB1*, *KIR2DL4*, *ZBTB16*, *EOMES*, *NKG7*, *TYROBP*, and *FCER1G*; and a naïve cell cluster expressing *PECAM1*, *TCF7*, *CD7*, *CCR7*, *SELL*, and *LEF1* (Supplementary Fig. S2). In terms of functional gene expression, these findings showed that circulating DP T cells have not only the characteristics of naïve cells but also of helper-, regulatory-, cytotoxic-, and innate-like cells.

### Circulating DP T cells exists as a separate population expressing naïve markers

**Figure 2:**
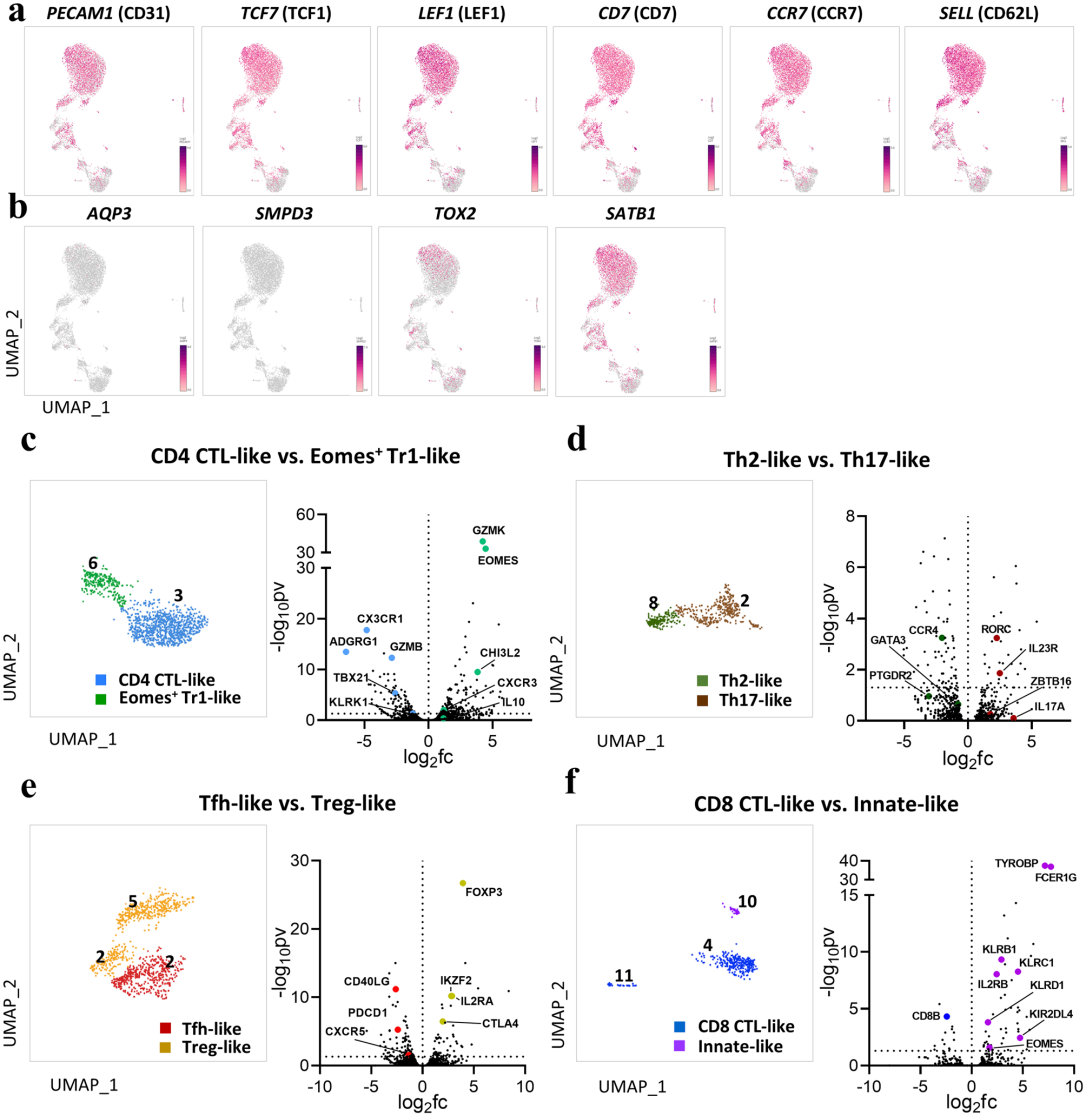
(**a,b**) Circulating DP T cells exist as a separate population expressing naïve markers. (**a**) UMAP representing naïve marker genes *PECAM1*, *TCF7*, *LEF1*, *CD7*, *CCR7*, and *SELL*. (**b**) UMAP representing marker genes associated with thymic development. *AQP3* and *SMPD3* are expressed at the immature DP stage, and *TOX2* and *SATB1* are expressed during DP-to-SP transition. (**c**–**f**) Identification of marker gene signatures for the eight defined clusters. UMAP of paired clusters and volcano plot showing the log (fold change) and − log (*p*-values) for pairwise differential expression between two DP T populations. (**c**) CD4 CTL-like *vs*. Eomes^+^ Tr1-like cells. (**d**) Th2-like *vs.* Th17-like cells. (**e**) Tfh-like *vs.* Treg-like cells. (**f**) CD8 CTL-like *vs*. Innate-like cells. All plots were generated using GraphPad Prism 8.0.2. The dotted horizontal line represents a *p*-value of 0.05. The number in UMAP indicates the cluster number.

The number of circulating DP T cells, which comprise about 2–4% of total T cells in the periphery^4^, increases with age^11^. As such, these populations are regarded as comprising mainly terminally differentiated effector/memory cells^8,10^, or as being derived from activated SP T cells^18^. However, UMAP visualization of DP single cells revealed a large clustering (clusters 0, 1, and 9) in which a substantial number of single cells shared similar genetic markers. That is, these clusters expressed naïve cell marker genes such as *PECAM1* (CD31), *TCF7* (TCF1), *LEF1*, *CD7*, *CCR7*, and *SELL* (CD62L) (Fig. 2a). To determine whether they are derived from a thymic T cell developmental stage, we identified expression of genes associated with the cell cycle. *AQP3* and *SMPD3*, both immature DP stage markers^19^, were lacking, but *SATB1* and *TOX2* (T cell development genes associated with DP-to-SP transition)^19,20^ were identified in the naïve clusters (Fig. 2b). In view of these findings, circulating DP T cells cannot be considered to be identical to immature stage DP thymocytes; thus these cells can be defined as a distinct subpopulation of naïve T cells.

### DP T subsets are characterized according to specific gene signatures

#### CD4 CTL-like and Eomes^+^ Tr1-like cells

Single-cell transcriptome profiling of clusters 3 and 6 revealed similar, albeit not identical, gene expression (Fig. 2c). Cluster 3 exhibited CD4 CTL-like cell-associated gene signatures with cytotoxic functions such as *CX3CR1*, *ADGRG1*, *KLRK1*, *TBX21,* and *GZMB*^21,22^. In contrast to these gene profiles, cluster 6 expressed Eomes^+^ Tr1-like cell-associated genes such as *EOMES*, *GZMK*, and *CHI3L2*, and lacked the canonical Treg markers *IL2R* and *FOXP3*^23^. Although this subset is associated with tumor progression, *IL10* expression by this cluster was limited, implying that its suppressive function is unclear. Given these findings, clusters 3 and 6 were defined as CD4 cytotoxic T lymphocytes-like cells and Eomes^+^ type 1 regulatory T-like cells, respectively.

#### Th2-like and Th17-like cells

Next, we focused on genes differentially expressed by two adjacent clusters. Both clusters showed gene expression patterns similar to those of helper T cell lineages (Fig. 2d). In cluster 8, Th2 cell-associated genes such as *GATA3*, *CCR4*, and *PTGDR2* were highly expressed^24^, but expression of the Th2 marker *IL4* was lacking. These results are consistent with the previous report that expression of the *IL4* gene in macaque monkeys is lower than that in humans^25^. By contrast, some cells in cluster 2 showed high expression of Th17-associated genes such as *RORC*, *CCR6*, *IL23R*, and *IL17A*^24^. Notably, *ZBTB16* (PLZF), which regulates and plays a critical role in the acquisition of the Th17 phenotype^26^, was identified in this cluster. Considering these gene expression patterns, we defined clusters 8 and 2 as Th2-like cells and Th17-like cells, respectively.

#### Treg-like and Tfh-like cells

We also identified scRNA-seq data that could be divided into two specific clusters (Fig. 2e). Cluster 5 and part of cluster 2 displayed high differential expression of genes associated with regulatory T cells. That is, the canonical gene signatures *FOXP3*, *IL2RA*, *CTLA4*, and *IKZF2* were clearly identified^24^. Therefore, we defined this single-cell cluster as Treg-like cells. In particular, this cluster could be distinguished from Eomes^+^ Tr1-like cells in cluster 6 with respect to *FOXP3* expression. We also found a distinct cluster expressing *CXCR5*, *PDCD1*, and *CD40LG*, along with homing receptors *CCR7* and *SELL*^27^. These are thought to be differentially expressed genes suitable for defining circulating follicular helper T cells.

#### CD8 CTL-like and innate-like cells

In the clusters with relatively small populations, we identified characteristics suggestive of mainly cytotoxic functions (Fig. 2f). These clusters (4, 10, and 11) shared the expression of *CRTAM*, *KLRD1*, *KLRK1*, *PRF1*, and *GZMB*^28^. Interestingly, we identified highly differentially expressed genes such as *EOMES*, and *ZBTB16* in a very small subset (cluster 10), suggesting a potential difference in function. In this cluster 10, *TYROBP* and *FCER1G*, which are associated with innate-like T cells^29^, were highly expressed, in addition to the killer cell-like receptor (KLR) family (e.g., *KLRC1* and *KLRB1*) and killer cell immunoglobulin-like receptor (KIR) family genes (e.g., *KIRD2DL4*)^16,29^. Based on these gene expression patterns, we defined clusters 4 and 11 as CD8 CTL-like, and cluster 10 as innate-like cells.

### Characterization of scRNA-seq by CD4 and CD8 feature barcodes

**Figure 3:**
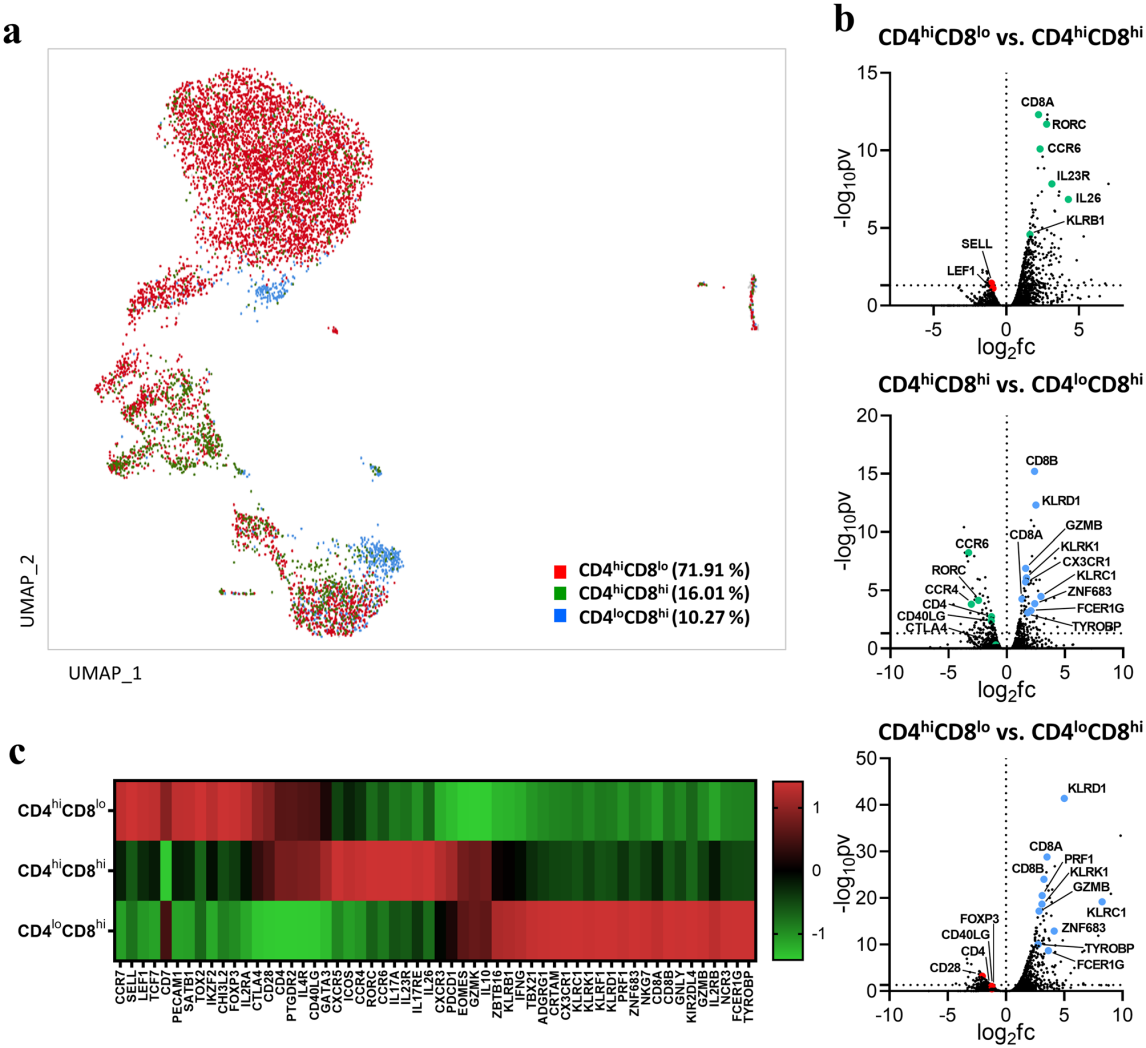
Characterization of scRNA-seq by CD4 and CD8 feature barcodes. (**a**) Distribution (cell composition) of CD4^hi^CD8^lo^, CD4^hi^CD8^hi^, and CD4^lo^CD8^hi^ cells based on ADT (CD4 and CD8α functional barcode) signals on UMAP plots (Loupe Browser 4.0.0). (**b**) Volcano plot showing DEG analysis among the CD4^hi^CD8^lo^, CD4^hi^CD8^hi^, and CD4^lo^CD8^hi^ fractions. (**c**) Heatmap showing relative gene expression for feature barcode levels using a Z-score obtained by DEG analysis. All volcano plots and heatmaps were generated using GraphPad Prism 8.0.2. The dotted horizontal line represents a *p*-value of 0.05. *ADT* antibody-derived tags.

To investigate whether the clustered subsets could be distinguished by ADTs, we performed cellular indexing of sorted DP T cells with CD4 and CD8α feature barcodes. Whole single cells were divided into three compartments according to the level of CD4 and CD8 coreceptor expression as follows: CD4^hi^CD8^lo^, CD4^hi^CD8^hi^, and CD4^lo^CD8^hi^ (Fig. 3a). Expression of the naïve marker genes mentioned above was predominant in the CD4^hi^CD8^lo^ subset but was also identified in the other two subsets. Comparison of each subset according to ADT levels revealed differences in expression of specific genes. That is, the CD4^hi^CD8^lo^ subset showed mainly naïve gene signatures, as well as expression of genes with regulatory (e.g., Eomes^+^ Tr-1-like and Treg-like) and helper (e.g., Th2- and Tfh-like) functions. Similarly, the CD4^hi^CD8^hi^ subset also exhibited expression of genes with helper and regulatory functions (e.g., Th2-, Th17-, Tfh-, and Treg-like), whereas the CD4^lo^CD8^hi^ subset showed expression of genes with cytotoxic and innate-like (e.g., CD8 CTL- and innate-like) functions (Fig. 3b,c). However, it was more difficult to distinguish some cell types, such as CD4 CTL-like cells. Taken together, the transcriptomic features of various DP T cell subsets can be distinguished according to the expression level of the CD4 and CD8 co-receptors.

### Heterogeneous subsets identified by scRNA-seq correspond to subsets identified by flow cytometry

**Figure 4:**
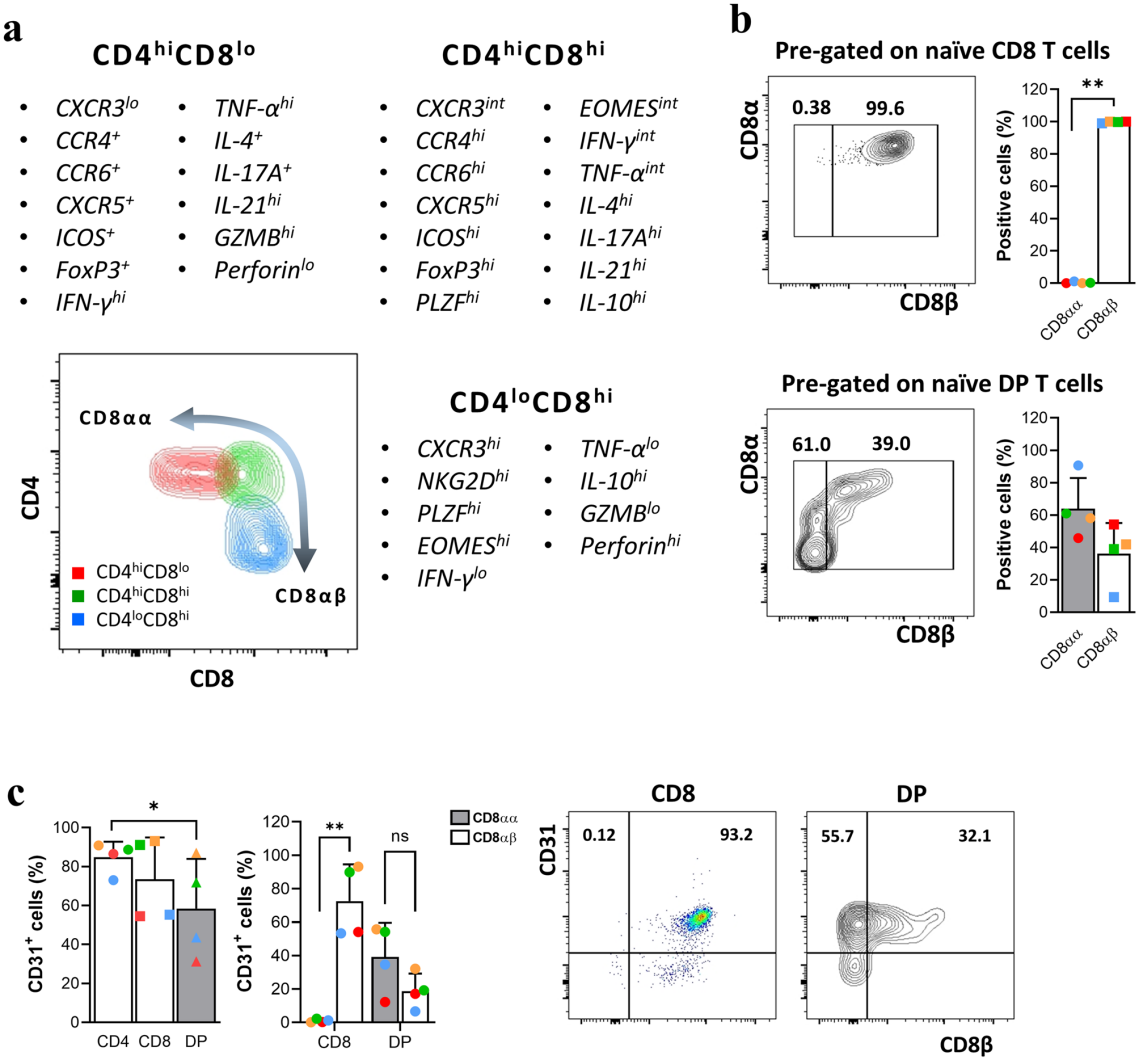
Phenotype and cytokine profile of each fraction of DP T cells, and differences in expression of CD8αα^+^, CD8αβ^+^, and CD31 by the naïve population. (**a**) Comparison of cell surface marker and cytokine expression among the CD4^hi^CD8^lo^, CD4^hi^CD8^hi^, and CD4^lo^CD8^hi^ fractions. (**b**) Percentage of CD8αα^+^ and CD8αβ^+^ cells within the naïve (CD28^+^CD95^−^) CD8 SP T and DP T cell populations. (**c**) Percentage of CD4, CD8, and DP T cells expressing CD31, and comparison of percentage of CD8αα^+^CD31^+^ and CD8αβ^+^CD31^+^ cells within the CD8 SP T and DP T cell populations. Data are expressed as the mean ± SDs (n = 4). Dot plots are representative of experimental data, all with similar results. Red dots on the bar graph represent data from the monkey used for scRNA-seq analysis. *p < 0.05; **p < 0.01. *ns* not significant, *GZMB* granzyme B.

To determine how closely the scRNA transcriptomic findings correspond with protein expression levels, we utilized flow cytometry to assess the phenotypic and cytokine production profiles of the CD4^hi^CD8^lo^, CD4^hi^CD8^hi^, and CD4^lo^CD8^hi^ fractions. Each DP fraction showed different expression of surface molecules, as well as production of different cytokines, according to expression of the CD4 and CD8 co-receptors (Fig. 4a). Expression of CD8αα was predominant in the CD4^hi^CD8^lo^ fraction, which comprised the majority of DP T cells. A previous report showed that the CD8αα homodimer is not expressed by naïve T cells^30^; thus we examined expression of the CD8 surface glycoprotein by CD28^+^CD95^-^ T cells. In contrast to all naïve CD8 SP T cells, which were CD8αβ, more than half of naïve DP T cells expressed CD8αα (Fig. 4b); they also showed high expression of CD31, much like conventional SP T cells. In addition, the coexpression of CD8αα and CD31 was high in DP T cells, unlike CD8 SP T cells (Fig. 4c).

**Figure 5:**
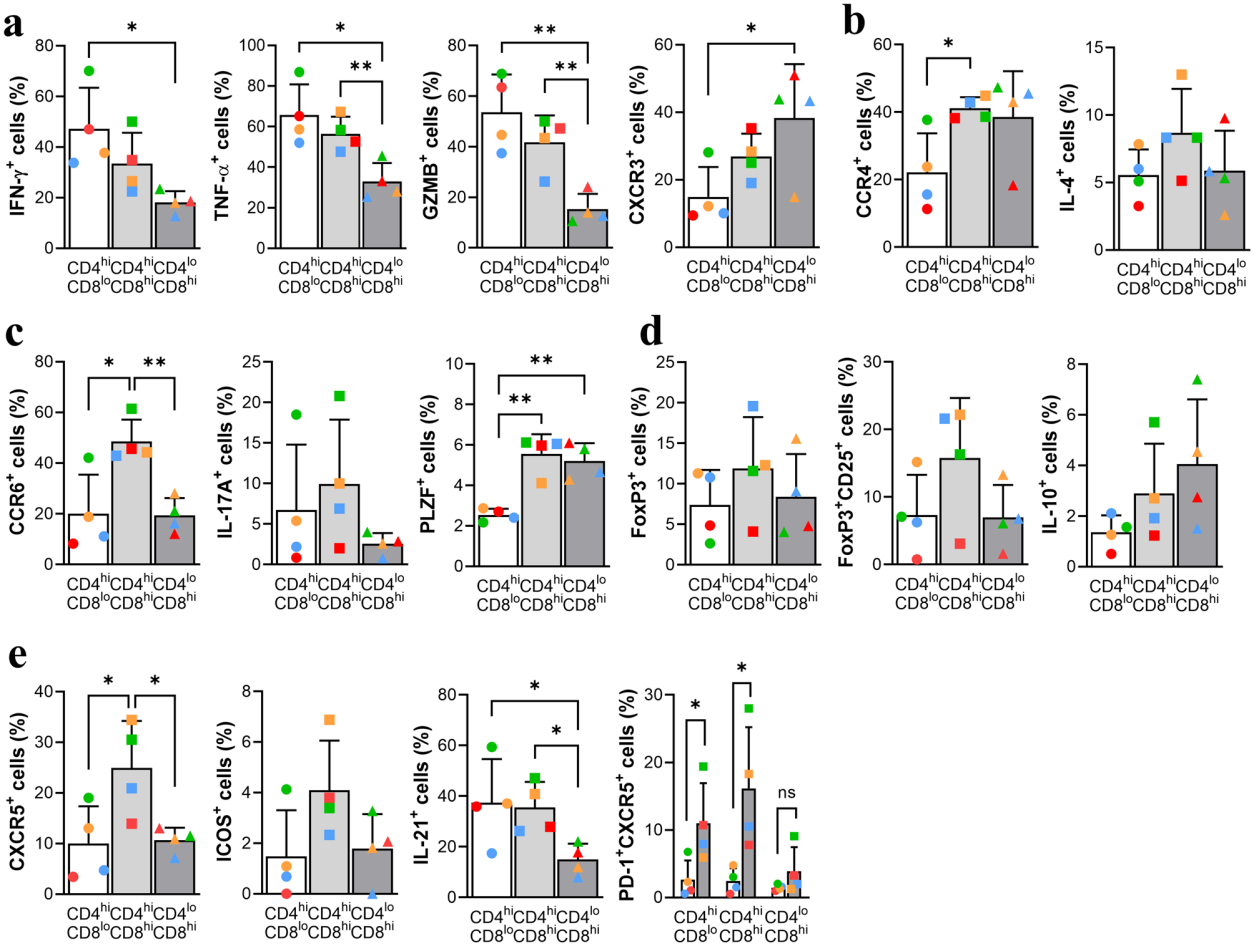
Phenotype and cytokine profile of the CD4^hi^CD8^lo^ and CD4^hi^CD8^hi^ fractions. (**a**) Percentage of cells expressing IFN-γ^+^, TNF-α^+^, Granzyme B^+^, and CXCR3^+^ within each DP T cell fraction. (**b**) Percentage of CCR4^+^ and IL-4^+^ cells within each DP T cell fraction. (**c**) Percentage of CCR6^+^, IL-17A^+^, and PLZF^+^ cells within each DP T cell fraction. (**d**) Percentage of FoxP3^+^, FoxP3^+^CD25^+^, and IL-10^+^ cells within each DP T cell fraction. (**e**) Percentage of CXCR5^+^, ICOS^+^, IL-21^+^, and PD-1^+^CXCR5^+^ cells before and after stimulation of PBMCs with plate-coated anti-CD3 2 μg/ml and soluble anti-CD28 2 μg/ml for 24 h. Cytokine production levels were measured after stimulation of PBMCs with PMA/ionomycin for 4 h. Data are expressed as the mean ± SDs (n = 4). Red dots on the bar graph represent data from the monkey used for scRNA-seq analysis. *p < 0.05; **p < 0.01; *ns* not significant.

In terms of phenotypic markers, the CD4^hi^CD8^lo^ subset produced significantly more CD4 CTL-associated cytokines (IFN-γ, TNF-α, and granzyme B)^21,31^ than the other subsets, but expression of Th1-associated CXCR3 was relatively low (Fig. 5a). The CD4^hi^CD8^hi^ subset expressed higher levels of Th2-associated markers such as CCR4 and IL-4^32^ than the other subsets (Fig. 5b). CCR6 and IL-17A, both associated with Th17 cells^33^, were highly expressed by this subset. Notably, expression of PLZF, which regulates and plays a critical role in acquisition of the Th17 phenotype in human cells^26^, was significantly high (Fig. 5c). Expression of FoxP3, a master regulator of Treg cells^17^, was relatively high in the CD4^hi^CD8^hi^ fraction, as was FoxP3/CD25 and IL-10 (Fig. 5d). Expression of CXCR5, a marker associated with circulating follicular helper T cells^34^, was significantly high in the CD4^hi^CD8^hi^ subset, as were ICOS and IL-21. In particular, PD-1/CXCR5 levels were upregulated in same fraction (CD4^hi^CD8^hi^) after stimulation with anti-CD3/CD28, as in a previous report^35^ (Fig. 5e). Overall, the results of phenotypic and cytokine profile analyses demonstrate that the DP subsets with high CD4 expression, that is, the CD4^hi^CD8^lo^ and CD4^hi^CD8^hi^ fractions, comprised cells exhibiting helper, regulatory, and cytotoxic functions similar to those of CD4 SP T cells.

**Figure 6:**
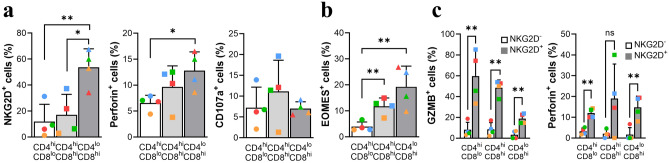
Phenotype and cytokine profile of the CD4^lo^CD8^hi^ fraction. (**a**) Percentage of NKG2D^+^, perforin^+^, and CD107a^+^ cells within each DP T cell fraction. (**b**) Percentage of EOMES^+^ cells within each DP T cell fraction. (**c**) Comparison of granzyme B^+^ and perforin^+^ cells between NKG2D^+^ and NKG2D^-^ DP T cell fractions. Cytokine production was measured after stimulation of PBMCs with PMA/ionomycin for 4 h. Data are expressed as the mean ± SDs (n = 4). Red dots on the bar graph represent data from the monkey used for scRNA-seq analysis. *p < 0.05; **p < 0.01; *ns* not significant, *GZMB* granzyme B.

By contrast, the CD4^lo^CD8^hi^ fraction showed obvious cytotoxic characteristics. NKG2D levels were significantly higher than those in the other two fractions (Fig. 6a). The CD4^lo^CD8^hi^ fraction also showed strong production of perforin, although there was no difference in CD107a expression compared with the other subsets (Fig. 6a). Expression of EOMES and PLZF, a transcription factor associated with innate-like CD8 T cells^16^, was also significantly high in the CD4^lo^CD8^hi^ fraction (Figs. 5c, 6b). In addition, measurement of cytotoxic enzymes such as perforin and granzyme B (Fig. 6c) showed the high cytotoxic potential in NKG2D^+^ cells in both the CD4^hi^ (e.g., CD4 CTL-like cells) and CD8^hi^ (e.g., CD8 CTL-like cells) subsets. However, despite identifying heterogeneous circulating DP T cells, it was more difficult to define Eomes^+^ Tr1-like cells due to co-production of IFN-γ and IL-10^36^ along with high expression of Eomes. Taken together, the results suggest that the CD4^lo^CD8^hi^ subset showing higher expression of CD8 than CD4 has a cytotoxic and innate-like phenotype and cytokine production profile.

## Discussion

The data presented herein demonstrate that circulating DP T cells comprise subpopulations with a variety of characteristics and functions. Compared with single-positive T cells, DP T cells exhibit pleiotropic features, including helper, cytotoxic, regulatory, and innate-like roles, depending on their expression of CD4 or CD8 co-receptors. These DP T cells can be developed by a variety of pathways, such as thymocyte-like DP T cells have observed in certain pathologic conditions (e.g., systemic sclerosis or atopic dermatitis)^37,38^.

In contrast to previous reports showing that DP T cells are derived from mature CD4^+^ T cells^39,40^ or CD8^+^ T cells^18^, our scRNA-seq data suggest the possibility that circulating DP T cells are derived from the thymus. First, we show that the three different cell clusters display representative naïve T cell markers (Fig. 2a,b), especially the CD4^hi^CD8^lo^ fraction; this is in contrast to the common belief that they comprise effector memory or terminally differentiated T cells^8^. Second, they may be distinct from thymic immature DP T cells in that they lack *AQP3* and *SMPD3*, known as thymic DP stage marker genes^19^. However, considering that they express *SATB1* and *TOX2*, which are expressed at the DP-to-SP transition stage^19^, it can be assumed that this population is derived incidentally from the thymus after positive and negative selection. Lastly, contrary to previous reports that CD8αα is never expressed on naïve T cells but is induced readily on strongly activated T cells^30,41^, our phenotypic analysis showed that many naïve DP T cells (CD28^+^CD95^−^) express CD8αα and high levels of CD31. Given these findings, we suggest that circulating DP T cells can be considered as a mature T cell lineage distinct from SP T cells.

Despite being a separate population, scRNA-seq and phenotypic analyses revealed that most DP T cells are similar to conventional CD4 or CD8 T cells. For example, the CD4^hi^CD8^lo^ and CD4^hi^CD8^hi^ subsets showed predominantly helper and regulatory function-associated gene signatures and phenotypes, much like differentiated CD4 T cells. As expected, a subset of the CD4^lo^CD8^hi^ fraction exhibited predominantly cytotoxic characteristics, much like those of CD8 CTLs. Additionally, CD4 CTL-like cells, which exhibit cytotoxic properties, were also identified in the NKG2D^+^ cells of the CD4^hi^CD8^lo^ fraction. Given these cytotoxic characteristics, these data support previous findings that peripheral DP cell populations are increased under chronic inflammatory conditions, such as transplantation^4^ and viral infection^9^, as well as in cancer patients^42^.

A recent report on bulk RNA sequencing of human thymic T cells^19^ identified unconventional T cell populations such as CD8αα^+^ T cells and Th17-like cells. Interestingly, our scRNA-seq data identified one cluster (cluster 10) that expressed CD8αα (Supplementary Fig. S3a) including the innate cell markers *EOMES*, *ZBTB16*(PLZF), *TYROBP*, and *FCER1G*^29^, suggesting that they are similar to unconventional CD8αα^+^ T cells of human^19^. Additionally, our identification of Th17-like cells (known as fetal-specific cells^19^), based on expression of *CD4*, *RORC*, *CCR6*, and *CD40LG*, may have revealed another type of unconventional T cell because they also express *KLRB1* and *ZBTB16*, both hallmarks of innate lymphocytes^43,44^. Meanwhile, we identified a subset that exhibits features different from those of regulatory T cells. Eomes^+^ Tr1 cells, characterized by expression of *GZMK* and *CHI3L2*, have been detected in the tumor microenvironment^23^. CHI3L2 belongs to a family of chitinase-like proteins (e.g., CHI3L1) reported to enhance inflammatory responses and promote tumor growth^45^, suggesting that Eomes^+^ Tr1-like DP T cells may be involved in tumor progression.

Among the remaining undefined clusters, cluster 12 did not match any immune-related terms in the gene ontology enrichment analysis, except for cell biology-associated gene signatures. Clusters 7 and 13 exhibited both T and B cell characteristics, that is, T cell marker genes such as *TRCA* (TCRα), *CD3E* (CD3ε), and *CD247* (CD3zeta) were clearly expressed, and B cell-associated gene signatures were identified simultaneously (Supplementary Fig. S3b). These subsets are presumed to be cells expressing dual receptors, which have been reported recently^46^.

Taken together, despite the limitation that the cell population was small, we were able to reveal the heterogeneity of DP T cells through scRNA-seq and flow cytometry analysis. We believe that these findings provide useful information that will increase our understanding of the relationship between circulating DP T cells and various immune-related diseases.

## Methods

### Subjects

Four healthy male cynomolgus monkeys (*Macaca fascicularis*) aged 2–10 years were used for the study. Blood samples were taken from the femoral vein. Isolated cells from blood were used for phenotyping and for the in vitro experiments. Single-cell RNA-seq analysis was performed on a sample from one monkey (10 years old). All animals were cared for in strict accordance with the National Institutes of Health Guide for the Care and Use of Laboratory Animals. This study was approved by the local Institutional Animal Care and Use Committee (IACUC) of Seoul National University Hospital (IACUC number: 20-0004-S1A0). All the experiments were performed in accordance with the relevant guidelines and regulations. All the experiments were performed in accordance with the ARRIVE guidelines.

### Cell preparation

Peripheral blood mononuclear cells (PBMCs) were isolated from heparin-anticoagulated whole blood using the Ficoll density gradient method (GE Healthcare, Uppsala, Sweden). All isolated cells were either used immediately for experiments or cryopreserved. If cryopreserved cells were used for experiments, they were thawed in a 37 °C water bath and then washed in RPMI 1640 (Biowest, Nuaillé, France) supplemented with 10% fetal bovine serum (FBS; Biowest). Then, the cells were resuspended in RPMI containing 10% FBS and cultured for 2–4 h in a 37 °C CO_2_ incubator to allow stabilization and recovery.

### Antibodies and flow cytometry

The following fluorochrome-labeled human monoclonal antibodies were used: CD3-PerCP-Cy™5.5 (SP34-2, BD Biosciences), CD4-PE/Cyanine7 (OKT4, BioLegend), CD8α-APC/Cyanine7 (SK1, BioLegend), CD8β-PE/Cyanine7 (SIDI8BEE, eBioscience, San Diego, CA, USA), CD20-FITC (2H7, BD Biosciences), CD28-PE (CD28.2, BD Biosciences), CD95-APC (DX2, BD Biosciences)¸ CD31-Pacific Blue™ (WM59, BioLegend), CXCR3-PE (1C6/CXCR3, BD Biosciences), CCR4-Brilliant Violet 421™ (L291H4, BioLegend), CCR6-APC (G034E3, BioLegend), CXCR5-PE (MU5UBEE, eBioscience), PD-1-APC (EH12.2H7, BioLegend)¸ ICOS-FITC (ISA-3, eBioscience), CD25-APC (M-A251, BioLegend), NKG2D-PE (1D11, BioLegend), PLZF-PE (Mags.21F7, eBioscience), EOMES-eFluor^®^ 660 (WD1928, eBioscience), FoxP3-PE (236A/E7, eBioscience), IL-4-Brilliant Violet 421™ (MP4-25D2, BioLegend), IL-10-APC (JES3-19F1, BD Biosciences), IL-17A-eFluor^®^ 660 (eBio64CAP17, eBioscience), IL-21-PE (3A3-N2, BioLegend), TNFα-Brilliant Violet 421™ (Mab11, BioLegend), IFNγ-PE (4SB3, BD Biosciences), Perforin-FITC (Pf-344, Mabtech), Granzyme B-FITC (REA226, Miltenyi Biotec), and CD107a-FITC (H4A3, BD Biosciences). For surface staining, prepared cells were resuspended in staining buffer (PBS, 0.5% BSA, and 0.5 mM EDTA), and single-cell suspensions were labeled with antibodies for 30 min at 4 °C. After surface staining, cells were washed and resuspended in staining buffer. For intracellular staining, surface-stained cells were washed with PBS prior to fixation and permeabilization using the FoxP3/Transcription factor staining buffer set (eBioscience). Then, intracellular cytokines and/or transcription factors were labeled with antibodies for 30 min at 4 °C. Flow cytometry was performed using a LSRFortessa X-20 (BD Biosciences) or LSRII (BD Biosciences) cytometer. All data were analyzed using the FlowJo software v10 (TreeStar, San Carlos, CA, USA).

### Cell stimulation and intracellular cytokine staining

Isolated cells were stimulated in two ways for phenotypic analysis and measurement of cytokine production capacity. After thawing as described above, cells were resuspended in RPMI/10% FBS, aliquoted (5 × 10^5^/200 μl per well) into 96-well plates, and allowed to stabilize for 2 h. For phenotypic analysis, PBMCs were stimulated with plate-coated anti-CD3 (2 μg/ml) and soluble anti-CD28 (2 μg/ml) in RPMI medium supplemented with 10% FBS and cultured for 24 h at 37 °C. The stimulated cells were washed and stained for surface proteins as described above. For analysis of cytokine production, PBMCs were stimulated with the Cell Stimulation Cocktail (plus protein transport inhibitors) (eBioscience). Next, intracellular cytokines were stained as described above.

### Sample preparation for single-cell RNA-seq

To prepare samples for scRNA-seq analysis, CD4^+^ T cells were separated from fresh PBMCs using the REAlease CD4 MicroBead Kit (Miltenyi Biotec). Next, CD4^+^CD8^+^ DP T cells were separated from isolated CD4^+^ T cells using the CD8 Microbeads Kit (Miltenyi Biotec). The isolated CD4^+^CD8^+^ DP T cells were then labeled with Totalseq-CD4 and Totalseq-CD8 antibodies: CD4, Clone SK3, Barcode sequence: GAGGTTAGTGATGGA (BioLegend); CD8, Clone SK1, Barcode sequence: GCGCAACTTGATGAT (BioLegend).

### Library construction and sequencing

Libraries were prepared using the Chromium controller according to the 10× Chromium Single-Cell V(D)J User Guide (10× Genomics). Briefly, cell suspensions were diluted in nuclease-free water to achieve a targeted cell count of 10,000. The cell suspension was then mixed with master mix and loaded, along with Single-Cell 5′ Gel Beads and Partitioning Oil, into a Single-Cell A Chip. RNA transcripts from single cells were uniquely barcoded and reverse-transcribed within droplets. Next, cDNA molecules were pooled and enriched by PCR. For the 5′ Gene Expression Library, the cDNA pool went through an end repair process (i.e., addition of a single ‘A’ base), followed by ligation of the adapters. The products were then purified and enriched by PCR to create the 5′ Gene Expression Library. The purified libraries were quantified by qPCR according to the qPCR Quantification Protocol Guide (KAPA) and qualified using Agilent Technologies 4200 TapeStation (Agilent Technologies). Finally, the libraries were sequenced using the HiSeq platform (Illumina) according to the read length in the user guide.

### Preprocessing and analysis of single-cell RNA-Seq data

The Cell Ranger v3.1.0 (10× Genomics) pipeline was used to generate FASTQ files from raw sequencing data for gene expression analysis of 5′ Gene Expression Library data, and for cell surface protein expression analysis in the feature barcode library. Illumina basecall files for each sample generated by the Illumina sequencing instrument were converted to the FASTQ format using the ‘mkfastq’ command. Gene expression libraries were analyzed using the ‘count’ command. Sequence reads were aligned to the *Macaca_fascicularis_5.0* genome reference for *Macaca fascicularis* using STAR (v2.5.1b) aligner. Feature barcoded libraries were matched to the target feature barcode reference. Next, gene and cell surface protein expression profiles for each cell were generated using information contained in the unique molecular identifier (UMI) and 10× cell barcode. Finally, cells were grouped into clusters according to gene expression.

### Advanced analysis of single-cell RNA-Seq data

The raw count matrices of 10× genomics were imported into Seurat 3.1.3. To select high-quality cells, cells with over 4000 or less than 200 unique feature genes and with a mitochondrial gene ratio of more than 20% were filtered out. After filtering, the count value of 13,738 genes across 8338 cells was normalized and scaled. Clustering and UMAP analysis were performed based on statistically significant principal components. Next, find significance markers for every cluster compared to all remains cells were determined using minimum fraction of min.pct cells and Wilcox rank sum test, report only the positive ones. Gene-Enrichment and Functional Annotation analysis of the significant probe list was performed using the g:Profiler tool (https://biit.cs.ut.ee/gprofiler/).

### Statistical analysis

Statistical analysis, except of the single-cell RNA-seq-data, was conducted using the Prism program (GraphPad Software, Inc., USA). All analytical data were tested for statistical significance using an unpaired *t*-test. A *p*-value < 0.05 was considered significant.

## Supplementary Information

Supplementary Figures.

## Data Availability

All scRNA-seq data are available on the Gene Expression Omnibus (GEO) with the accession number GSE183835.
