# Supplementary material for: Cellular heterogeneity of circulating CD4+CD8+ double-positive T cells characterized by single-cell RNA sequencing
Source: Sci Rep. 2021 Dec 8;11:23607. doi: 10.1038/s41598-021-03013-4 (PMC8655006; doi:10.1038/s41598-021-03013-4)
Supplement: Supplementary file 1 — Supplementary Figures. [file 41598_2021_3013_MOESM1_ESM.pdf]

Cellular heterogeneity of circulating $CD4^+CD8^+$ double-positive T cells characterized by single-cell RNA sequencing

Sung Min Choi[1], Hi Jung Park[1], Eun A Choi[1], Kyeong Cheon Jung[2,3,4], and Jae Il Lee[2,5,*]

Supplementary Figure S1

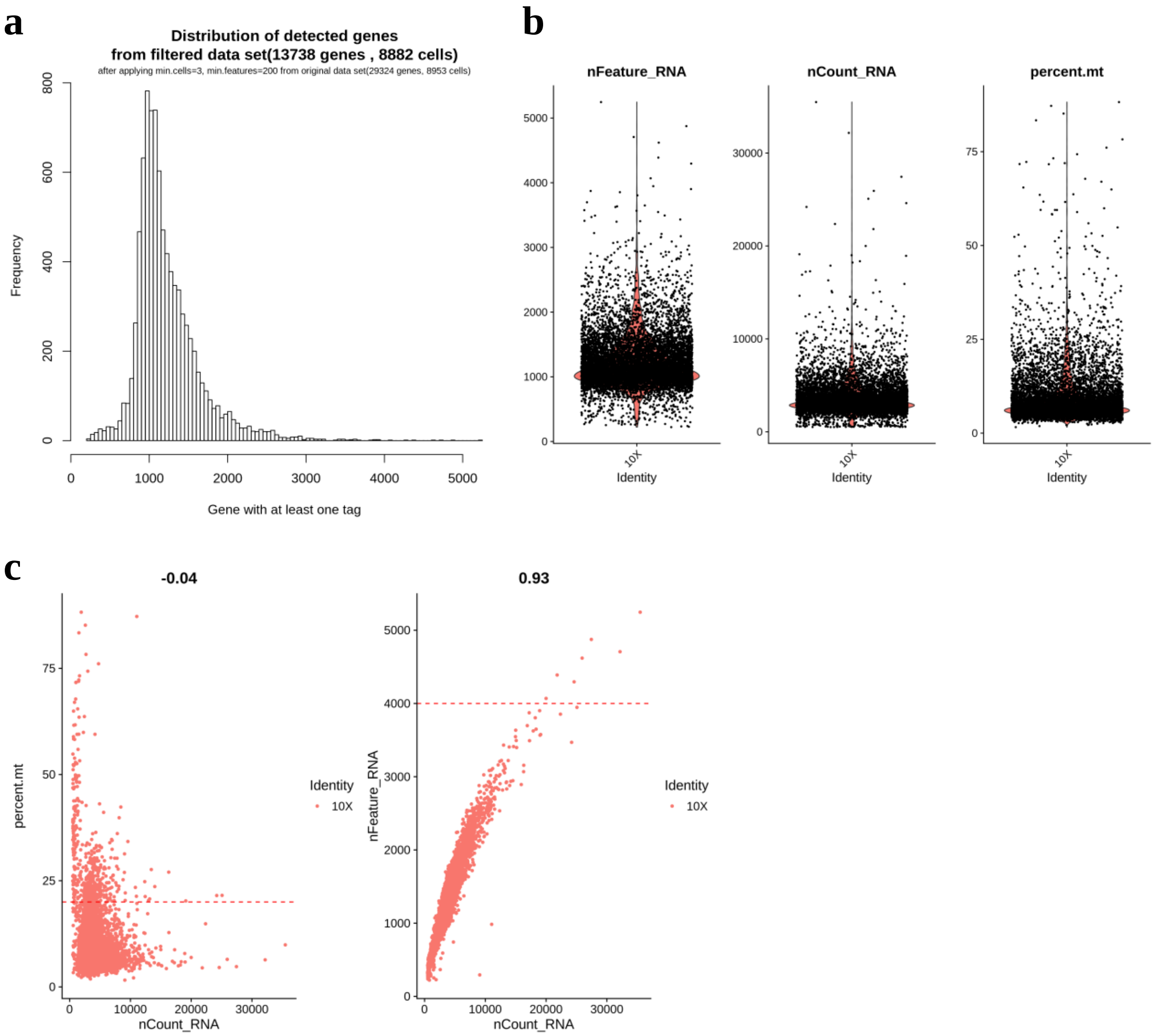

S1. Quality control result for scRNA-seq analysis. (a) The histogram showing the frequency of cells containing genes with at least one UMI after filtering out cells/features from original data set. (b) The violin plots representing the distribution of nGene/nUMI/percentage of mitochondrial gene for each cell. (c) The scatter plots representing relationships of mitochondrial percentage-molecule counts and genes-molecule counts for each cell.

Supplementary Figure S2

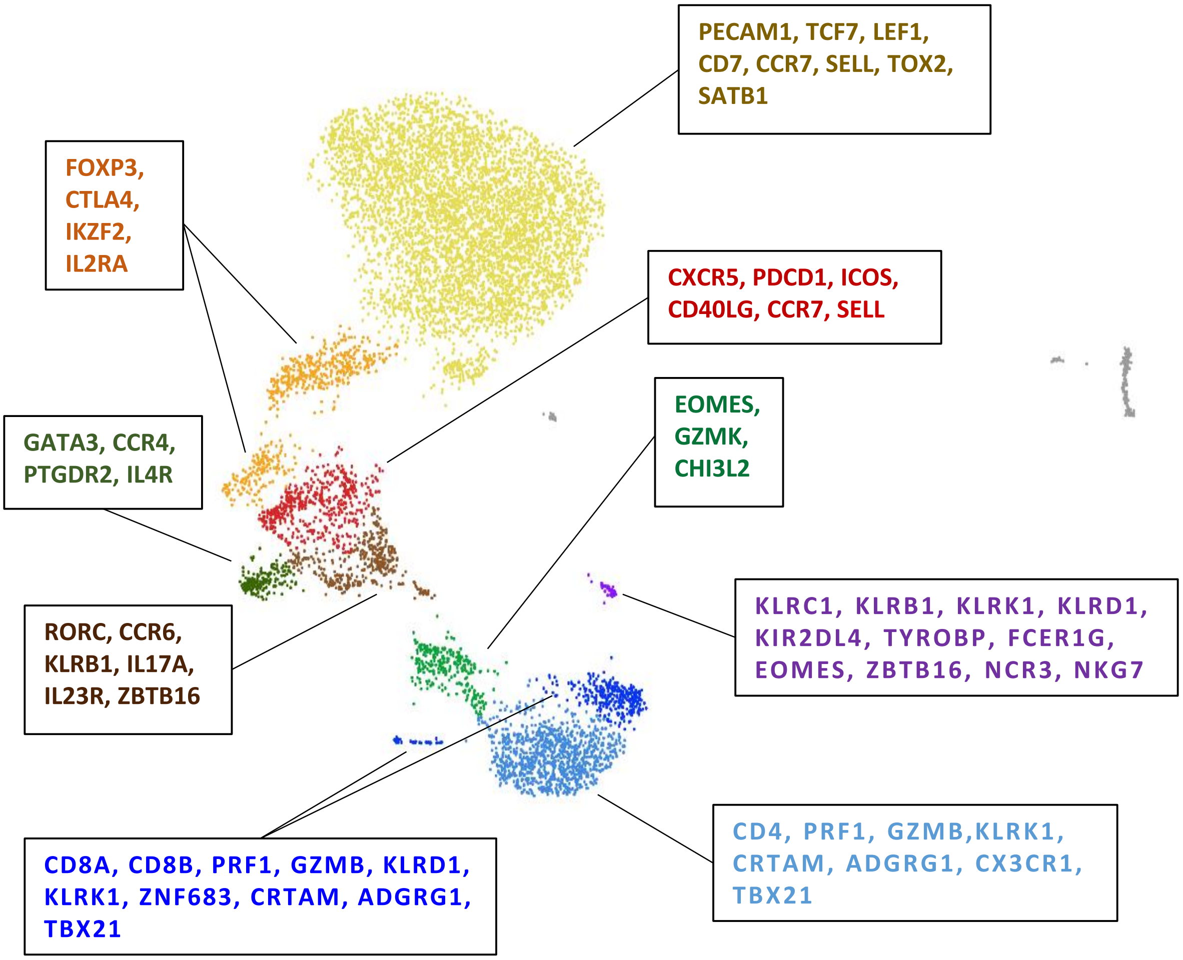

S2. Differential expression of genes that define heterogeneous clusters of $CD4^{+}CD8^{+}$ DP T cells.

Supplementary Figure S3

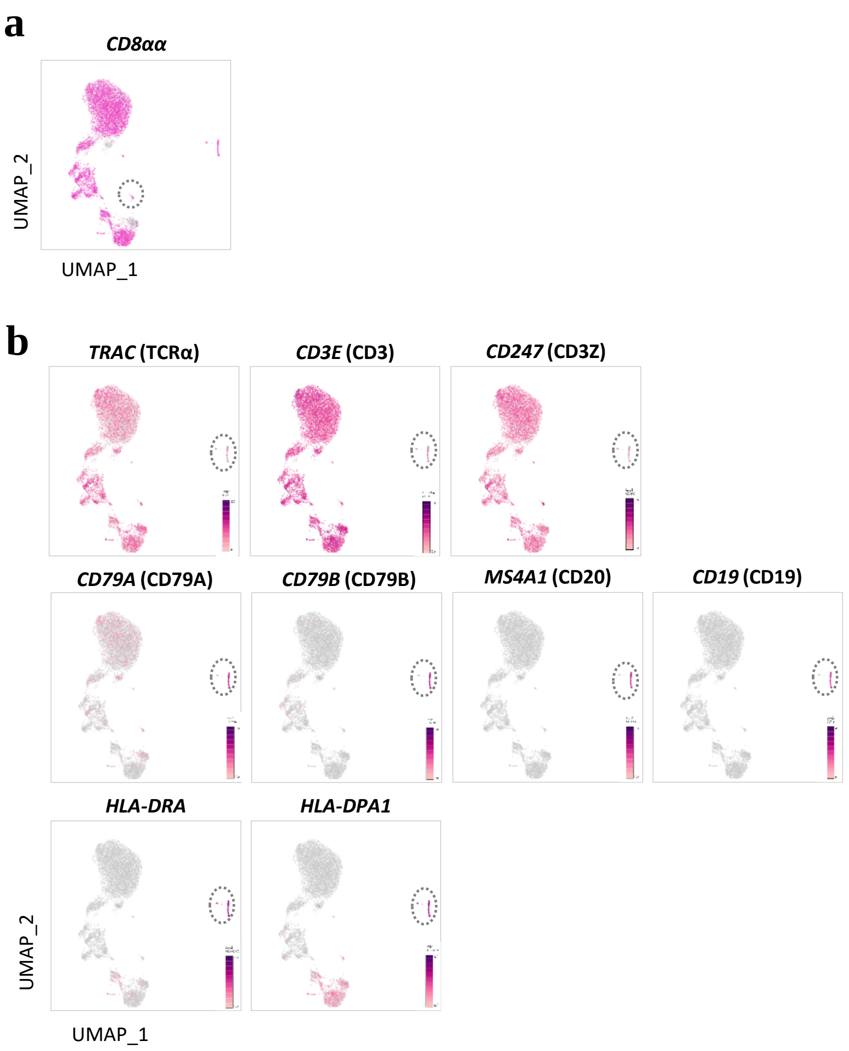

S3. (a) UMAP representing *CD8αα*. (b) UMAP representing T and B cell-associated genes.
